# Dark Triad Psychopathy Outperforms Self-Control in Predicting Antisocial Outcomes: A Structural Equation Modeling Approach

**DOI:** 10.3390/ejihpe12060041

**Published:** 2022-05-27

**Authors:** Pedro Pechorro, Shelby Curtis, Matt DeLisi, João Maroco, Cristina Nunes

**Affiliations:** 1 Faculdade de Psicologia (The Center for Research in Neuropsychology and Cognitive and Behavioral Intervention (CINEICC), Psychological Assessment and Psychometrics Laboratory), University of Coimbra, 3000-115 Coimbra, Portugal; ppechorro@gmail.com; 2 Psychology Research Centre (CIP), Universidade do Algarve, 8005-139 Faro, Portugal; 3 Social Psychology Ph.D. Program, University of Nevada, Reno, NV 89557, USA; srcurtis@nevada.unr.edu; 4 Department of Sociology, Iowa State University, Ames, IA 50011, USA; delisi@iastate.edu; 5 William James Centre for Research, ISPA-Instituto Universitário, 1149-041 Lisbon, Portugal; jpmaroco@ispa.pt

**Keywords:** aggression, conduct disorder, dark triad, juvenile delinquency, self-control, crime seriousness

## Abstract

Dark Triad traits and self-control are considered viable causal precursors to antisocial and criminal outcomes in youth. The purpose of the present study is to concurrently compare how Dark Triad traits and self-control differ in terms of predicting self-reported juvenile delinquency, CD symptoms, proactive overt aggression, and crime seriousness. The sample consisted of 567 (M = 15.91 years, SD = 0.99 years, age range = 14–18 years) Southern European youth from Portugal. Structural-equation-modelling procedures revealed that the psychopathy factor of Dark Triad traits presented the strongest significant hypothetical causal associations with the antisocial/criminal outcomes, followed by self-control. Machiavellianism and narcissism presented the lowest causal associations. Our findings indicate that psychopathy, as operationalized in the Dark Triad, concurrently surpasses self-control and the remaining factors of the Dark Triad in terms of predicting antisocial/criminal outcomes in youth. This suggests that behavioral disinhibition, or a core incapacity to regulate one’s conduct, is central for understanding delinquency and externalizing psychopathology. Comparatively, the interpersonal component of dark personality features, such as Machiavellianism and narcissism, are secondary for understanding crime.

## 1. Introduction

Delinquent behaviors often manifest prior to adulthood. These behaviors are generally limited to adolescence, but a minority of delinquent youth persist in antisocial or criminal behaviors throughout the life span [1,2,3]. Thus, many researchers aim to understand the social factors and personality traits that differentially predict persistent engagement in antisocial behavior. Gottfredson and Hirschi’s [4] general theory of crime proposes that a lack of self-control predisposes people towards antisocial and criminal behaviors. Other perspectives move beyond self-control disposition and articulate that specific personality features increase involvement in conduct problems. For example, personality features such as psychopathy, Machiavellianism, and narcissism have also been associated with antisocial behaviors, to varying degrees [5,6,7]. In particular, psychopathy is one of the best clinical predictors of violent-crime recidivism [8,9]. Although low self-control and Dark Triad [7] features overlap, they are distinct individual difference markers [10,11] that independently predict antisocial or criminal outcomes [12]. In this study, we investigate the relative strength of the causal associations between self-control, Dark Triad traits, and assorted antisocial outcomes.

In their general theory of crime, Gottfredson and Hirschi [4] argue that self-control can explain all delinquent and criminal behavior, and that all other associations with such behavior are spurious and are just other outcomes of low self-control. This theory postulates that self-control reflects a hedonic orientation to maximize pleasure and avoid pain. Popular theories of self-control suggest that there are four primary domains that control thoughts, emotions, impulses, and performance [13,14]. High self-control can be perceived of as the ability to adapt and fit the self with the environment, and to refrain from behaving in socially undesirable ways [15]. Therefore, low self-control, particularly in the performance domain, should be associated with engagement in socially undesirable behaviors, which would include criminal activity.

Studies on the relationship between self-control and crime have indeed found consistent significant associations [16,17,18,19]. The meta-analysis by Pratt and Cullen [17] suggests effect sizes of 0.26–0.28 in low self-control predicting crime. However, this effect size decreased when studies were longitudinal, which suggests that self-control might not be as effective in predicting persistent crime. This suggests that the link between self-control and general antisocial behavior could be attributable to conceptually similar but distinct constructs. For instance, Friehe and Schildberg-Hörisch [20] found evidence to suggest that the link between self-control and crime is due primarily to increased risk taking, rather than to engagement in antisocial behavior. However, longitudinal research on adolescents suggests that low self-control is associated with higher levels of aggressive and delinquent behavior [21]. Thus, other constructs appear to coexist with self-control in the etiology of conduct problems.

### 1.1. Interrelations between Self-Control and the Dark Triad

Despite Gottfredson and Hirschi’s [4] claim that other associations with crime beyond self-control are spurious, other researchers have found evidence to challenge their general theory. In particular, the Dark Triad of personality has been long associated with antisocial behavior and criminal activity [6,7]. The Dark Triad, which consists of traits that share a common core of callous manipulation, is comprised of Machiavellianism, narcissism, and psychopathy [7]. Each of these traits have been individually associated with criminal outcomes. Narcissism is characterized by increased grandiosity, a sense of entitlement, and a sensitivity to ego threat [22]. Machiavellianism is characterized by a cynical worldview, a long-term focus, and strategic flexibility [23]. Psychopathy is characterized by high dysfunctional impulsivity, behavioral disinhibition, and aggression [10,24,25,26]. With respect to criminal behavior, narcissism and Machiavellianism have been studied most often for instances of white-collar and financial crime [27,28], whereas psychopathy is highly predictive of generalized criminal behavior [29,30,31], and especially of violent crime [8,32,33,34]. All three are related to distinct types of aggression and antisocial behavior in adolescents [35], and to bullying behaviors in adults [5].

In adolescents, all three Dark Triad traits are differentially associated with antisocial behaviors. Whereas Machiavellianism was strongly associated with emotional dysregulation, it was not uniquely predictive of delinquency [35]. In the same study, both psychopathy and narcissism predicted overt aggression and delinquency. In a study on cyber-aggression in adolescents, Pabian and colleagues [36] found that only psychopathy was significantly predictive of cyber-aggression. Other research also finds support for Dark Triad traits, and especially psychopathy, in predicting delinquent behaviors and adolescent aggression [37,38,39]. Therefore, these traits present an alternate perspective on the causal precursors to antisocial and criminal behavior.

Both the Dark Triad traits and self-control are viable causal precursors to antisocial and criminal outcomes in adolescents. However, this may be due, in part, to overlap between the constructs. Psychopathy, in particular, is consistently related to low self-control and high levels of dysfunctional impulsivity [10,11]. When correlated with the Big Five traits of personality, both sets of constructs have similar associations with low conscientiousness, high extraversion, and low agreeableness [40,41].

Some research on self-control, the Dark Triad, and crime suggests that they are additively predictive. Narcissism and low self-control were both independently and interactively predictive of violence in an adult sample [42], as were psychopathy and low self-control in adolescents [43]. In a study on both substance use and criminal offending, the Dark Triad and self-control were both additively predictive of offending, but only self-control independently predicted substance use [44]. DeLisi and colleagues [45] found that, in a head-to-head test, low self-control was associated with more forms of delinquency than psychopathy and was a stronger independent predictor of chronic self-control. However, Wright and colleagues [12] found that the Dark Triad outperformed self-control in predicting violent delinquency, and that the variables significantly interacted. Thus, there is mixed evidence as to the nature of the relationship between self-control, the Dark Triad, and criminal outcomes.

### 1.2. Current Aim

Some theories of crime suggest that self-control is the only causal precursor to criminal behavior [4]. However, personality research suggests that the Dark Triad of personality might be an equally strong, if not stronger, predictor of antisocial and criminal outcomes. The present study aimed at comparing how Dark Triad traits and self-control concurrently predict self-reported delinquency, conduct-disorder symptoms, proactive overt aggression, and crime seriousness among Southern European male and female youth. On the basis of previous research, we hypothesized that both self-control and the measures of the Dark Triad, and particularly psychopathy, would be independent predictors of these outcomes, although we did not make specific hypotheses about the relative strength of these predictors.

## 2. Materials and Methods

### 2.1. Participants

The community sample consisted of 567 youth (M = 15.91 years, SD = 0.99 years, age range = 14–18 years), and was subcomposed of females (*n* = 256, M = 15.80 years, SD = 1.02, range = 14–18) and males (*n* = 311, M = 15.99 years, SD = 0.96, range = 14–18). When comparing females and males, no significant differences were detected in terms of age (F = 3.38, *p* = 0.06), socioeconomic status (U = 38,318.5, *p* = 0.41), or education (F = 0.63, *p* = 0.42). The majority of the participants were Portuguese nationals (88.4%) with approximately nine years of education, on average (M = 8.95, SD = 0.94).

### 2.2. Measures

*Sociodemographic questionnaire.* This questionnaire was elaborated with the aim of describing the participants’ characteristics, including nationality, sex, age, education, and socioeconomic status (SES) (estimated by considering the parents’ formal education and job, a method that provides three ordinal levels: high, medium, and low).

### 2.3. Predictors

*Dirty Dozen* (DD) [10]. This is a brief 12-item tridimensional measure of the Dark Triad construct of personality composed of Machiavellianism (e.g., “I have used deceit or lied to get my way”; “I have used flattery to get my way”), psychopathy (e.g., “I tend to be unconcerned with the morality of my actions”; “I tend to be callous or insensitive”), and narcissism (e.g., “I tend to want others to admire me”; “I tend to want others to pay attention to me”) factors. Items are rated on an ordinal Likert scale (ranging from Strongly Disagree to Strongly Agree). The score of each of the factors can be obtained by adding the respective items, and a total score can also be obtained. Higher scores indicate higher levels of Dark Triad traits (i.e., Machiavellianism, psychopathy, and narcissism). The version of the DD validated in Portugal among the youth population was used with a 5-point ordinal scale [46]. The internal consistency for the current study was Narcissism α = 0.88, Psychopathy α = 0.93, Machiavellianism α = 0.86, and DD total α = 0.93.

*Brief Self-Control Scale* (BSCS) [14]. This is a brief 13-item self-report unidimensional measure of self-control. The BSCS includes items such as “I refuse things that are bad for me”, “I am able to work effectively toward long-term goals”, and “Sometimes I can’t stop myself from doing something”. Items are rated on a 5-point Likert scale (ranging from 1 = Not at all like me to 5 = Very much like me). The total score of the BSCS can be obtained by adding the items. The appropriate items were reverse scored so that higher scores reflect lower levels of self-control. The version of the BSCS validated in Portugal among the youth population was used in the current study [47]. The internal consistency for the current study, estimated by Cronbach’s alpha (α), was BSCS α = 0.93.

### 2.4. Outcomes

*Add Health Self-Report Delinquency* (AHSRD) [48]. This is a 17-item self-report measure of juvenile delinquency, originally developed for the National Longitudinal Study of Adolescent Health (Add Health in its abbreviated form). The AHSRD taps criminal behaviors that occur during the last year before the assessment (e.g., “Take something from a store without paying for it”; “Steal something worth less than €50). Items are rated on a 4-point Likert scale (ranging from 0 = None to 3 = Five or more times). The total delinquency score can be obtained by adding the items. Higher scores indicate higher levels of self-reported criminality. The AHSRD was validated in Portugal among the youth population [48]. The internal consistency for the current study was AHSRD α = 0.93.

*Conduct Disorder Screener* (CDS) [49]. This is a brief 6-item self-report screener created to identify adolescents with CD. The items (e.g., “I got into fights; I skipped school”; “I got into trouble for lying or stealing”) are intended to be representative of CD diagnosis according to the APA’s (1994) DSM-IV, and they are rated on a 4-point ordinal Likert scale (ranging from 1 = Rarely or none of the time to 4 = Most or all of the time). Higher scores indicate higher levels of CD symptoms. The version of the CDS validated in Portugal among the youth population was used [50]. The internal consistency for the current study was CDS α = 0.84.

*Peer Conflict Scale-20* (PCS-20) [51]. This is brief 20-item self-report four-dimensional measure that taps the different forms and functions of aggression. The PCS-20 has five proactive overt (PO) items (e.g., “I start fights to get what I want”), five proactive relational (PR) items (e.g., “I gossip about others to become popular”), five reactive overt (RO) items (e.g., “When someone hurts me, I end up getting into a fight”), and five reactive relational (RR) items (e.g., “If others make me mad, I tell their secrets”). Items are rated on a 4-point Likert scale (ranging from 0 = Not at all true to 3 = Definitely true). The score of each factor can be obtained by adding the respective items, and a total score can also be obtained by adding all the items. Higher scores indicate higher levels of aggression. The PCS-20 was validated in Portugal among the youth forensic population [52]. The internal consistency for the current study was PO α = 0.90, PR α = 0.82, RO α = 0.92, RR α = 0.84, and PCS-20 total α = 0.94.

*Crime Seriousness.* This variable was measured using a Portuguese version of the Delinquency Seriousness Classification Index (DSCI), originally developed by Loeber and colleagues [53]. The DSCI employs a four-level progressive ordinal sequence, with higher scores indicating higher seriousness levels of crimes committed by youth, such as violent-felony offenses.

### 2.5. Procedures

The Ministry of Education (ME) of Portugal was contacted in order to obtain authorization to assess the female and male participants of the present study. These participants were attending randomly selected schools in the southern regions of Portugal, including the capital Lisbon and the city of Faro in the Algarve region. Written parental authorization was previously obtained, and then the potential participants were themselves informed about the aims of our investigation and were asked to collaborate voluntarily and anonymously. Because of various motives, some youth were excluded (e.g., those who could not read Portuguese, those who were reluctant to participate). We also inspected each questionnaire visually and excluded incomplete questionnaires and suspiciously completed questionnaires (e.g., items systematically responded to in the same direction without paying attention to positive or negative wording). The rate of participation was 89%. No form of compensation was given, including monetary compensation. The measures and sociodemographic questionnaire included in the present study were administered in small groups of participants.

### 2.6. Data Analysis

EQS 6.4 [54] structural-equation-modeling software was used to estimate models (namely, to analyze the hypothetical causal associations of the predictors with the outcomes). We tested four models. In Model 1, Dark Triad traits and self-control causally predicted self-reported delinquency. In Model 2, Dark Triad traits and self-control causally predicted CD symptoms. In Model 3, Dark Triad traits and self-control causally predicted proactive overt aggression. In Model 4, Dark Triad traits and self-control causally predicted crime seriousness. Four items per measure with the highest loadings were selected to build the latent measurement models, and no modification indices were used to improve these models. Maximum Likelihood Robust (MLR) methods with covariance matrices were used; these methods work well when distributions are not severely non-normal (absolute skewness and kurtosis values below 3 and 10, respectively) [55]. The following goodness-of-fit indices served to evaluate the different models: Satorra–Bentler chi-square/degrees of freedom (SBχ2/df), Comparative Fit Index (CFI), Root Mean Square Error of Approximation (RMSEA), and Incremental Fit Index (IFI). The following criteria were considered for an adequate fit: SBχ2/df < 5, CFI and IFI > 0.90, RMSEA < 0.08; and for a good fit: SBχ2/df < 2, CFI and IFI > 0.95, RMSEA < 0.06 [55,56].

IBM SPSS Statistics v27 [57] software was used to examine descriptive statistics, Pearson correlations, group differences, and the reliability of the measures (i.e., Cronbach’s alpha). Pearson correlations were considered high if above 0.50, low if below 0.20, and moderate if in between. To compare the female and male groups, ANOVAs and Mann–Whitney’s U test were used. Alpha coefficient was considered adequate if above 0.70, and good if above 0.80 [55].

## 3. Results

Table 1 presents the Pearson correlation matrix of the measures that were used in the present study. The correlations between the measures were high (i.e., above 0.50) and statistically significant, except for the correlation between self-control and narcissism, which was moderate.

**Figure 1:**
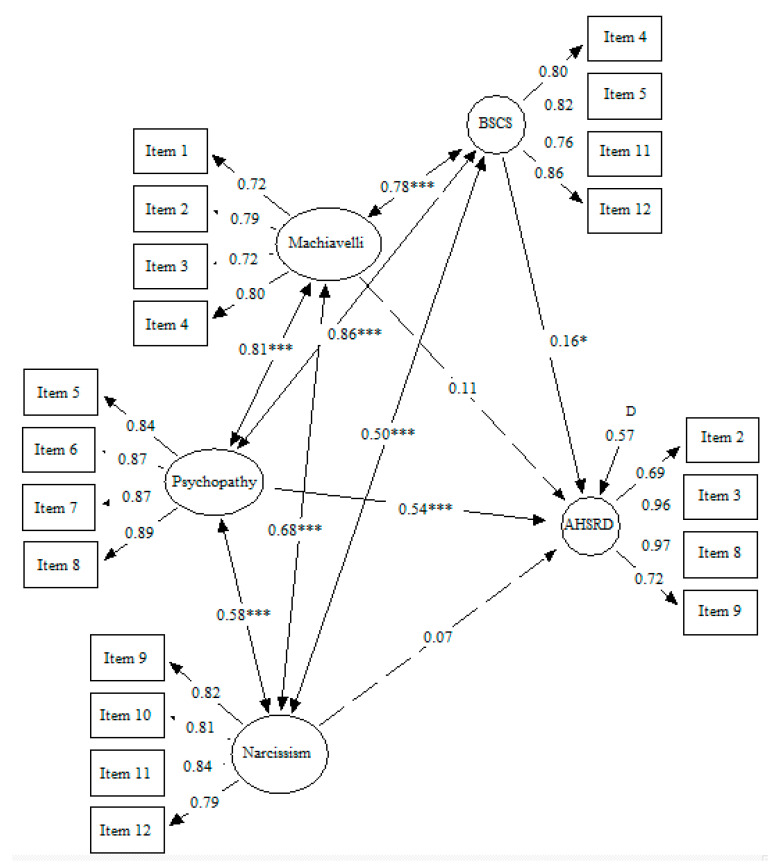
Dark Triad traits and low self-control predicting delinquency. *Note.* Machiavelli: Machiavellianism; BSCS: Brief Self-Control Scale: AHSRD: Add Health Self-Report Delinquency; * *p* ≤ 0.05; *** *p* ≤ 0.001.

Figure 1 displays the first model. This model presented an adequate fit: SBχ2/df = 4.05, CFI = 0.93, IFI = 0.93, RMSEA = 0.07 [0.06–0.08]. Psychopathy and self-control positively and significantly predicted the self-reported delinquency outcome. However, the Machiavellianism and narcissism prediction over the self-reported delinquency did not reach statistical significance. Psychopathy presented the highest regression coefficient (β = 0.54, *p* < 0.001), followed by self-control (β = 0.16, *p* < 0.05).

**Figure 2:**
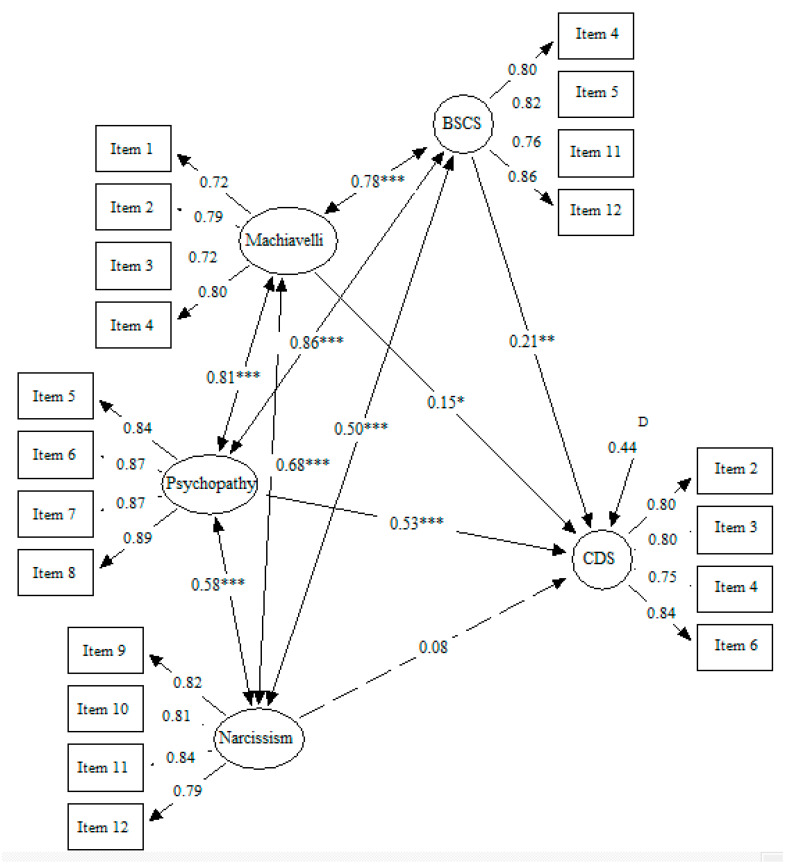
Dark Triad traits and low self-control predicting CD symptoms. *Note.* Machiavelli: Machiavellianism; BSCS: Brief Self-Control Scale; CDS: Conduct Disorder Screener; * *p* ≤ 0.05; ** *p* ≤ 0.01; *** *p* ≤ 0.001.

Figure 2 shows the second model. This model presented an adequate fit: SBχ^2^/df = 3.88, CFI = 0.93, IFI = 0.93, RMSEA = 0.07 [0.06–0.08]. Machiavellianism, psychopathy, and self-control positively and significantly predicted the CD-symptoms outcome, but not narcissism. Psychopathy presented the highest regression coefficient (β = 0.53, *p* < 0.001), followed by self-control (β = 0.21, *p* < 0.05), and then Machiavellianism (β = 0.15, *p* < 0.05).

**Figure 3:**
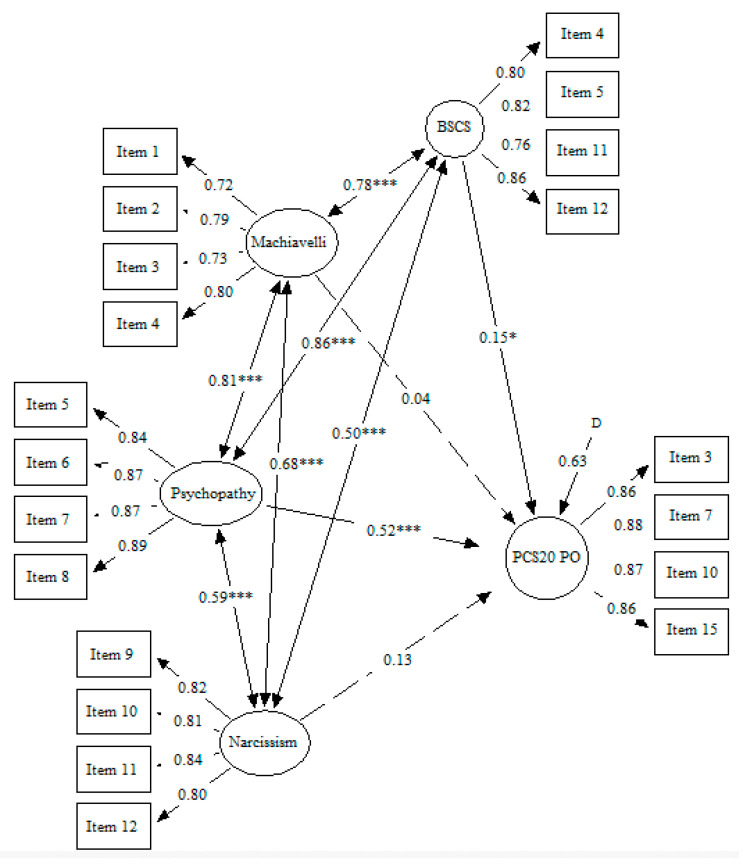
Dark Triad traits and low self-control predicting proactive overt aggression. *Note.* Machiavelli: Machiavellianism; BSCS: Brief Self-Control Scale; PCS20 PO: Brief Peer Conflict Scale 20 Proactive Overt Aggression; * *p* ≤ 0.05; *** *p* ≤ 0.001.

Figure 3 presents the third model. This model presented an adequate fit: SBχ^2^/df = 4.26, CFI = 0.92, IFI = 0.92, RMSEA = 0.07 [0.07–0.08]. Psychopathy and self-control positively and significantly predicted the proactive-overt-aggression outcome, but Machiavellianism and narcissism did not. Psychopathy presented the highest regression coefficient (β = 0.52, *p* < 0.001), followed by self-control (β = 0.15, *p* < 0.05).

**Figure 4:**
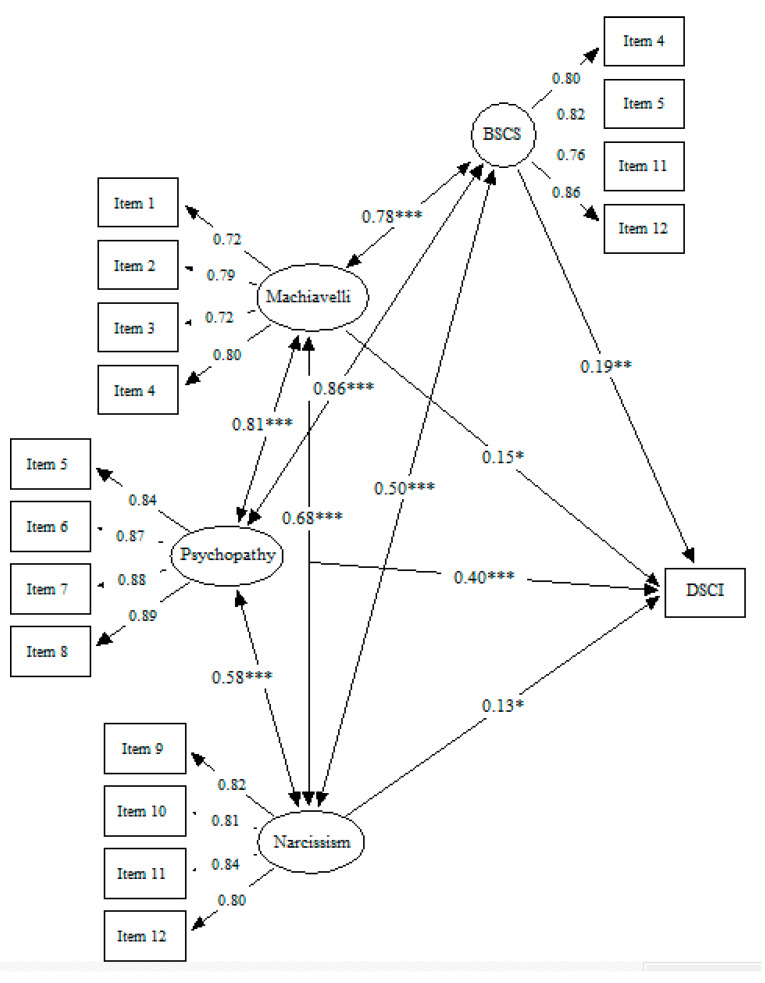
Dark Triad traits and low self-control predicting crime seriousness. *Note.* Machiavelli: Machiavellianism; BSCS: Brief Self-Control Scale; DSCI: Delinquency Seriousness Classification Index; * *p* ≤ 0.05; ** *p* ≤ 0.01; *** *p* ≤ 0.001.

Finally, Figure 4 shows the fourth model. This model presented an adequate fit: SBχ^2^/df = 4.82, CFI = 0.93, IFI = 0.93, RMSEA = 0.08 [0.08–0.09]. The four predictors positively and significantly predicted the crime seriousness. Psychopathy presented the highest regression coefficient (β = 0.40, *p* < 0.001), followed by self-control (β = 0.19, *p* < 0.01), then Machiavellianism (β = 0.15, *p* < 0.05), and lastly narcissism (β = 0.13, *p* < 0.05). Additionally, it is important to mention that the effects that are reported in the four models take into consideration the fact that the four predictors (i.e., Machiavellianism, psychopathy, narcissism, and self-control) are positively and significantly associated (*p* ≤ 0.001).

## 4. Discussion

The present study investigates the relative strength of the causal associations between the Dark Triad (i.e., psychopathy, Machiavellianism, and narcissism), low self-control, and antisocial/criminal outcomes. Dark Triad traits and low self-control are relevant constructs to the study of antisociality and criminality that present distinct individual difference markers [7,10]. During the last decade, several relevant studies have demonstrated the significant influence of both psychopathy and self-control as consistent and robust predictors of psychopathology and antisocial conduct [16,18,19,30,32,58,59]; however, studies that concurrently examine Dark Triad traits and low self-control among youth are much scarcer.

More specifically, the present study compared how the Dark Triad and self-control were associated with self-reported juvenile delinquency, CD symptoms, proactive overt aggression, and crime seriousness among Southern European youth. Our findings demonstrate that Dark Triad psychopathy presented the strongest significant causal associations with the several antisocial/criminal outcomes among the four structural equation models that were examined, followed by self-control. The Machiavellianism factor and the narcissism factor of the Dark Triad always presented the lowest causal associations, and they failed to reach statistical significance in most of the models examined. Our findings have several theoretical implications.

First, the findings of the present study are consistent with Wright and colleagues’ [12] investigation that found that the Dark Triad outperforms self-control in predicting violent delinquency. The findings also conflict with other studies that show that low self-control is associated with more forms of delinquency than psychopathy, and that only self-control independently predicts substance use, compared to the Dark Triad features [44,45]. Prior research used a regression-based framework that lacks the complexity and sequencing of variables that structural equation modeling offers. It is important to mention that the present study adds to the literature by using structural equation models to examine the data that offer more meaningful results in terms of validity, reliability, and the complex patterns of relationship analysis [60,61].

Second, the findings bear on important theoretical debates about general theories and the relative empirical strength of individual-level factors and their associations with crime. General theorists that advocate for self-control [4] or psychopathy [24,25,29] are supported by copious research findings that show that these individual-level factors are integral to conduct problems. Although these constructs have coexisted for decades, they have only relatively recently started to be integrated into criminological studies. The balance of which individual-level factor is “better” is uncertain, with some studies showing that self-control and psychopathy are coequal predictors of crime, while other studies favor one construct over the other [12,44,62,63,64,65]. We join that dialogue by showing that both psychopathy and self-control are significantly associated with various forms of externalizing conduct; however, psychopathy is the strongest predictor.

Third, in the event that psychopathy is the indispensable individual-level predictor of crime and analogous outcomes, this also suggests that the Dark Triad model is redundant, in that narcissism and a calculating, manipulative interpersonal style that is similar to Machiavellianism is already part of the disorder. Conceptually, it does not make much sense to “add on” additional dark personality features to a psychopathy condition that is replete with dark personality features. We concur with prior researchers who similarly express concern that the Dark Triad features are not sufficiently distinct [58,66], even with the more recent Dark Tetrad, which adds a sadism facet [67], and the Dark Core, which expands it even further [68].

Fourth, behavioral disinhibition, or a core incapacity to regulate one’s conduct, is central for understanding delinquency and other externalizing psychopathologies. This suggests that the presentation style in the interpersonal component of dark personality features, such as narcissism and Machiavellianism, are secondary or superfluous for understanding crime. We suspect that the egocentric features that are inherent to low self-control [4] and psychopathy pertain more to a basic drive to satisfy one’s wants and desires at the expense of or indifference to others. This egocentric drive or self-centeredness may not involve a cajoling, calculating, ingratiating style, which would support the weaker effects for narcissism and Machiavellianism. Indeed, among youth who are perpetrating the most serious forms of delinquency, and whose conduct rises to the level of clinical behavioral disorder, narcissism and calculated manipulation pale to brute antisocial force.

The limitations of the current investigation can inform future research and can serve to contextualize the findings. The study has a correlational/nonexperimental basis, and we did not experimentally investigate the real causal relationships between the variables. Moreover, it is a cross-sectional study, and, because of that, it is not possible to ascertain the temporal ordering of the effects, or to examine the long-term associations between the study variables. This is an important and challenging area of research because both self-control [69,70,71] and psychopathy [72,73,74] are moderately to highly heritable and thus have a substantial genetic etiological basis. This means that it is difficult to establish which behavioral tendencies emerge first, which influence the others, and which relate to conduct problems. We suspect that psychopathy is a more acute psychological condition relative to self-control, which is more normative. Thus, other investigators can model whether self-control deficits increase psychopathic functioning, whether these effects are reciprocal across developmental periods, and whether these constructs continue to influence the others vis-à-vis conduct problems over time.

The current findings also likely reflect shared method variance, as all outcomes are self-reported. Although meta-analytic research supports the use of self-reports for the Dark Triad [58], external data sources are always useful to compare to how participants view themselves. Additionally, our investigation was conducted among a school sample, which does not allow for the generalization of our findings to other types of samples, such as justice-involved or at-risk-for-delinquency youth. The severity and mix of self-control deficits and psychopathic features are much greater among justice-involved youth [45,75]; thus, future studies should be conducted among forensic samples (e.g., detained youth offenders, youth in confinement facilities, clinic-referred children and adolescents with significant behavioral disorders) to potentially replicate the current work. Finally, it is important to recognize that narcissism is a multifaceted construct that can have positive associations with productive prosocial conduct [76,77,78], which may explain some of the weaker effects in the current models.

## 5. Conclusions

One of the important aims of criminology is to influence the capacity to deter future delinquent and antisocial behavior on the basis of scientific findings that can guide the design of preventive and early intervention strategies among youth. We hope the present study contributes to the incentivization of further research on the self-control and the Dark Triad construct among Southern European youth to deter the development of conduct problems and other related psychosocial problems.

## Figures and Tables

**Table 1:** Pearson correlation matrix.

	1	2	3	4	5	6	7	8
1 Machiavellianism	-							
2 Psychopathy	0.71 ***	-						
3 Narcissism	0.60 ***	0.54 ***	-					
4 BSCS	0.68 ***	0.78 ***	0.45 ***	-				
5 AHSRD	0.61 ***	0.77 ***	0.50 ***	0.68 ***	-			
6 CDS	0.64 ***	0.77 ***	0.52 ***	0.70 ***	0.82 ***	-		
7 PCS-20 PO	0.60 ***	0.71 ***	0.51 ***	0.65 ***	0.83 ***	0.74 ***	-	
8 DSCI	0.64 ***	0.72 ***	0.53 ***	0.68 ***	0.82 ***	0.83 ***	0.73 ***	-

*Note.* BSCS: Brief Self-Control Scale; AHSRD: Add Health Self-Report Delinquency; CDS: Conduct Disorder Screener; PCS-20 PO: Brief Peer Conflict Scale Proactive Overt Aggression; DSCI: Delinquency Seriousness Classification Index; *** *p* < 0.001.

## Data Availability

Data are available upon reasonable request.
